# Effect of illite pretreatment on germinated Brown rice with Special Reference to amino acids, antioxidants, texture, and mineral elements

**DOI:** 10.1016/j.heliyon.2024.e28843

**Published:** 2024-04-09

**Authors:** Dong-Heun Han, Hwa-Jin Kim, So-Hyun Kim, Il-Doo Kim, Arjun Adhikari, Jeong-Ho Kim

**Affiliations:** a Illite Team, Economy Division, Yeongdong-gun Office, South Korea; b School of Applied Biosciences, Kyungpook National University, Daegu, South Korea; c International Institute of Research and Development Kyungpook National University, Daegu, South Korea; d Department of Green Technology Convergence, Konkuk University, Chungcheongbuk-do, 27478, South Korea

**Keywords:** illite, Germination, Quality characteristics, antioxidant activity, brown rice

## Abstract

The pretreatment process of various foods has been reported to improve their nutritional properties. The soaking of brown rice improves the texture and nutrients, which are crucial for cooking and maintaining its high functional value. Illite, a clay mineral, has recently been discovered to improve the nutritional value of seeds. Based on these findings, we soaked brown rice with different concentrations of illite solution for different durations and allowed the germination to perform analyses. Soaking the brown rice for 6 h with a germination period of 48 h was determined to be the optimal condition because of its higher sprout length. In addition, this optimal condition had improved textural characteristics such as reduced hardness, gumminess, chewiness, and cohesiveness, and it also had increased adhesiveness and stabilized resilience and springiness. The treatment solutions were free from heavy metal contaminants, whereas the mineral contents such as K, Ca, Fe, Mg, and Na were significantly increased with the increase in illite concentration. Moreover, our results showed that illite treatment could preserve the color appearance and seed germination. The ratio of essential amino acids to non-essential amino acids and antioxidants (phenolic contentγ-oryzanol, and flavonoid) of germinated brown rice was considerably increased with illite treatment. In germinated brown rice, an increase in DPPH and superoxide dismutase levels, a slight decrease in flavonoids, and no difference in polyphenol content were observed. These findings suggest that pre-soaking brown rice seeds with the appropriate concentration of illite could enhance their nutritional properties, which might attract consumers' interest to include this in their daily diet.

## 1 Introduction

*RICE* is part of more than half of the human population's lifestyle and has shaped the economies, diets, and cultures economies of millions of people [[1], [2], [3]]. There are about 8000 rice varieties, each with its own unique qualities and nutritional value. Following the post-harvest process, all rice varieties can be classified as either brown or white [4,5]. White rice (WR) has been the dominant cereal grain for consumption for several decades. Approximately 739.1 million metric tons of rice were produced in paddy fields around the world, and after milling, 490.5 million metric tons of WR were produced. Recently, there has been increased interest in studies regarding the eating habits related to brown rice (BR) [6]. BR is recognized as a substantial source of nutrients; however, it retains some of its bran layers and the embryo, which has a bitter taste and takes longer to boil. Therefore, convincing people to rather eat BR is challenging because of its taste, which is less pleasant compared to that of WR [7].

BR has been reported to contain a high nutrient content, including minerals, unsaturated fatty acids, amino acids, phenolic compounds, antioxidants, and **γ**-oryzano [8]**.** In addition, BR has been proven to contain anti-diabetic bioactive substances [9]. A switch from WR to BR is anticipated to help reduce the risk of type 2 diabetes, however is a difficult task to convincethe rice-eating population [10]. Reports have indicated that parboiled germinated brown rice (PGBR)-exposed rats exhibited reduced cardiac lesions, such as cardiomyopathy, hypertension, oxidative stress, and liver injury [11]. In addition to having anti-diabetic properties, eating BR grains has been shown to have anticancer, antihyperlipidemic, antihypertensive, and anti-cardiovascular disease effects [12].

The development of BR-related products and their consumption may have a significant impact on health, the economy, and the environment. During the last decade, approximately 49 products related to germinated brown rice (GBR) have been patented [13]. It has also been reported in bread that is prepared from GBR flour, which is gluten free and has a higher protein content, lipids, and bioactive compounds with increased antioxidant activity and reduced phytic acid [14]. A common strategy to increase the value of seeds is to treat them before or during germination, e.g., BR's iron concentration was increased when it was fortified with ferrous sulfate during germination [15]. In addition, treatment with ascorbic acid improved the quality and digestibility of BR [16].

Several innovative processing techniques to alter the physicochemical properties of wholegrain BR (*Oryza sativa* L.) have been developed, such as non-thermal techniques, thermal treatments, high hydrostatic pressure, irradiation, pulsed electric fields, cold plasma, ultrasound, heating pretreatment, and drying techniques [12]. However, a simple, innovative method that could be accessed by a layperson to improve the quality of BR is lacking. Therefore, we hypothesized that the application of illite, a clay mineral that contains several different mineral elements, may significantly enhance the growth, germination, and mineral nutrients of BR based on the previous experiment by Ha et al. [17]. There are limited reports on how illite affects plant growth and development or how it improves the germination and nutritional quality of seeds. Considering the benefits of GBR and the significance of illite in improving sprout quality, this study investigated the biochemical properties of GBR, such as antioxidants, amino acids, color, dietary fiber, and mineral nutrients, when treated with illite.

## 2 Materials and methods

### 2.1 Preparation of GBR

#### 2.1.1 Determination of optimal condition for soaking and germination

BR *(O. sativa* L.) that was obtained from the Korean rice cultivar Ilpum Byeo was used in this study. Illite powder was obtained from Yeongdonggun, Chungcheong-bukdo, Korea. One kilogram of BR was soaked in normal distilled water for 0, 3, 6, and 24 h and kept in a netted bag covered with towel, incubated at (30 °C ± 2 °C; relative humidity (RH): 60 %), and allowed to germinate for 72 h. During the course of germination, at every 4 h interval, each of the bag was dipped for 1 h in normal distilled water. The sprout germination was observed every day and the optimal condition (6 h of soaking and germination for 48 h: dipping in water for 1 h/4 h interval) was selected. A sprout length of 0.5–1 cm was detected at this optimal condition, which was almost similar to that of 72 h **(Supplementary File: Time** Table 1, Table 2**).**

**Table 1:** Hunter's color values of germinated brown rice treated with different concentrations of illite.

Sample	Color value		
	L^＃^ (Lightness)	a^＃^ (Redness)	b^＃^ (Yellowness)
**IBR-0**	85.28 ± 0.59a	1.09 ± 0.11a	9.78 ± 0.28a
**IBR-1**	85.49 ± 0.89a	1.03 ± 0.21a	9.72 ± 0.57a
**IBR-3**	85.19 ± 0.79a	1.08 ± 0.13a	9.80 ± 0.38a
**IBR-5**	85.26 ± 1.09a	1.04 ± 0.25a	9.77 ± 0.64a

L^**＃**^, Lightness (100, white; 0, black); a^**＃**^, redness (−, green; +, red); b^**＃**^, yellowness (−, blue; +, yellow). ^3^ Values represent the mean ± standard deviation of three replicates. Different letters in the same column express significant differences at p < 0.05 (Tukey test).

**Table 2:** Mineral contents (mg/kg of dry weight) of germinated brown rice cultivated after soaking the seed in different concentrations of illite.

Element	Sample			
	IBR-0	IBR-1	IBR-3	IBR-5
**Ca**	276.87 ± 7.52d	334.92 ± 7.16b	308.19 ± 7.49c	398.91 ± 3.24a
**Cu**	5.85 ± 0.04d	9.60 ± 0.07a	5.93 ± 0.01c	6.87 ± 0.01b
**Fe**	10.16 ± 0.19d	11.49 ± 0.11c	13.52 ± 0.16b	17.16 ± 0.13a
**K**	1145.33 ± 52.06BCE	1072.49 ± 27.99	1211.73 ± 49.23b	1413.46 ± 48.99a
**Mg**	824.52 ± 33.20b	839.17 ± 18.72b	882.02 ± 30.38b	1030.90 ± 8.56a
**Mn**	21.61 ± 0.23c	21.78 ± 0.22c	23.15 ± 0.21b	27.40 ± 0.23a
**Na**	149.19 ± 6.44b	140.37 ± 3.08b	148.69 ± 6.26b	168.51 ± 8.21a
**Zn**	20.19 ± 0.31b	19.79 ± 0.17b	21.63 ± 0.14a	22.49 ± 0.20a
**Total**	2453.72	2449.61	2614.86	3085.70

Each data point represents the mean ± standard deviation of at least three replicates. Different letters in the same row denote significant differences at p < 0.05 (Tukey test).

#### 2.1.2 Illite treatment experiment

Based on the determined optimal conditions, the BR was treated with three different concentrations (1, 3, and 5 %, w/v) of illite solution, including a control (0 %, water only), at 25 °C for 6 h. After 6 h of soaking, the moistened BR samples were kept into netted plastic bags and incubated at 30 °C for 48 h. During the 48 h of incubation, the germinating BR was moistened every 1 h by briefly dipping them into their respective solutions (0, 1, 3, and 5 %) that were used for soaking. The GBR samples were classified according to the concentration of illite powder used as the soaking solution for the BR: IBR-0: GBR soaked only with water; IBR-1: GBR soaked with 1 % illite powder; IBR-3: GBR soaked with 3 % illite powder; and IBR-5: GBR soaked with 5 % illite powder. The freshly harvested GBR samples were kept at −70 °C for 24 h and then freeze-dried samples. The lyophilized samples were ground into powder using a commercial grinder (HIL-G-501, Hanil Co., Seoul, Korea) and filtered through a 100-μm mesh sieve. The pulverized samples were kept in airtight sample bottles and stored at −20 °C until analysis. The mineral element content of the treated water control (0 %, normal distilled water) and illite (1, 3, and 5 %, w/v) was determined by inductively coupled plasma mass spectrometry (ICP-MS) (ICP AES: Varian Vista, Victoria, Australia).

### 2.2 Color measurement of BR

The color of BR was measured according to the method described by Kim et al. [18]. In brief, the L^**＃**^ (lightness), a^**＃**^ (redness, + or greenness, -), and b^**＃**^ (yellowness, + or blueness, -) values of the sprout powder were determined using a Chroma meter (CR-300, Minolta Corp., Osaka, Japan). A Minolta calibration plate (YCIE = 94.5, XCIE = 0.3160, YCIE = 0.330) and a HunterLab standard plate (L^**＃**^ = 97.51, a^**＃**^ = −0.18, b^**＃**^ = +1.67) were used to standardize the instrument using a D65 illuminant.

### 2.3 Determination of mineral content

The mineral content of the GBR samples was measured according to the method described by Skujins [19], with a few modifications. Half a gram of rice powder was suspended in 15 mL of nitric acid and diluted with an equal volume of distilled water. The concentration of the mineral elements was measured using an ICP-MS (ICP AES: Varian Vista).

### 2.4 Quantification of free amino acid content

The free amino acids were determined according to the protocol described by Mun et al. [20]. In brief, 1.5 g of freeze-dried pulverized sample was extracted with 10 mL of ice-cold 6 % (v/v) perchloric acid, incubated for 30 min on ice, and centrifuged at 4600×*g* for 15 min. The supernatant was separated, filtered, and adjusted to pH 7.0 using a KOH solution (33 %, w/v), and centrifuged again at 4600×*g* for 10 min to remove the potassium perchlorate precipitate. The pH of the supernatant was adjusted to 2.2 with 10 M HCl, and the final volume was brought up to 50 mL with distilled water. A 2 mL sample aliquot was mixed with 1 mL of lithium citrate buffer (pH 2.2), and the obtained solvent was injected into an automatic amino acid analyzer (Biochrom-20, Pharmacia Biotech Co., Uppsala, Sweden).

### 2.5 Analysis of antioxidant activities (DPPH, SOD, polyphenol and flavonoids)

The sample was extracted with absolute methanol (1:10; 1 g sample in 10 mL of methanol) by suspending a mixture in a shaking incubator (140 rpm, 25 °C) for 8 h. The extract was then vacuum-filtered, centrifuged at (3000 rpm, 15 min), filtered by a 0.2-μm syringe filter (Waters Co., Milford, MA, USA), and used for analysis of each of the parameter. All the absorbances were measured using a microplate spectrophotometer (Multiskan GO, Thermo Fisher Scientific, Finland).

The DPPH free radical-scavenging potential of the sprouts was assessed following the method described by Blois [21]. The extract was then 100 μl of extract and 0.1 % (w/v) DPPH methanolic solution were combined in microplate wells and kept in dark condition for 30 min at 25 ± 2 °C and the absorbance was measured at 517 nm.

The SOD-like activities of the GBR samples were determined following the analysis procedures described by Debnath et al. [22]. The amount of oxidized pyrogallol during the reaction was estimated by measuring the absorbance value of the mixture at 420 nm.

The Polyphenolic Content was quantified following the Folin-Ciocalteau method described by Dhungana et al. [23]. The absorbance value was measured at 750 nm. Gallic acid (GA) was used as the standard to plot the calibration curve, and the total phenolic content of the GBR samples was determined as a GA equivalent (GAE).

The flavonoid content of the GBR was estimated following the method described by Zhishen et al. [24], with some modifications. The absorbance value was measured at 510 nm. Quantification was performed by the calibration curve plotted using quercetin (QE) as a standard.

### 2.6 Determination of γ-oryzanol contents

The γ-oryzanol content was determined following the method described by Xu and Godber [25]. In brief, 5 g of GBR was mixed with 20 mL of ethyl acetate and 20 mL of dichloromethane. The mixture was vortexed and stirred at 200 rpm at 25 °C for 1 h and then centrifuged at 10,000 rpm at 15 °C for 10 min. The supernatants were separated, and the amount of γ-oryzanol was determined using a high-performance liquid chromatography (HPLC) system (Agilent, Waldbronn, Germany Zorbax Eclipse XDB-D18 column (150 mm × 4.6 mm in diameter)). The mobile phase was methanol/acetonitrile/dichloromethane/acetic acid (50:44:3:3, v/v). The isocratic flow rate was 1 mL/min, and the UV detector was set at 315 nm. Sample volumes of 5 μL were used for the sample injection. The limit of detection and limit of quantification values were calculated by multiplying 3.3 and 10 by the standard deviation of the intercept, respectively.

### 2.7 Phytate (phytic acid) extraction and quantification

The determination of the total phytate content was performed according to the method described by Carlson et al. [26]. In brief, 0.5 g of lyophilized pulverized samples of GBR were suspended in 20 mL of 0.5 M HCl and stirred for 3 h. The solution was then centrifuged at 2100 × g for 20 min and filtered. The final obtained supernatant was analyzed for total phytate content using an HPLC system (Agilent).

### 2.8 Textural analysis of GBR

The texture analysis of GBR was performed using a Brookfield texture analyzer (CT3 4500, Middleboro, MA 02346, USA). GBR at the optimal conditions (6 h soaking and germinating for 48 h) was kept in a cylindrical-shape container (D × H, 50 × 5 mm), wrapped in plastic film, and subjected to 20 °C for 10 min. The texture profile analyses were performed using a compression flat-head plunger under the following conditions: pre-test speed of 1.0 mm/s; test speed of 0.5 mm/s; and return speed of 0.5 mm/s. The parameters analyzed at room temperature were hardness (g), adhesiveness (mJ), cohesiveness, springiness (mm), gumminess (g), and chewiness (mJ). These parameters were obtained using Micro Stable Software (England) from the average of measurements performed in triplicate.

### 2.9 Statistical analysis

Data were subjected to analysis of variance (ANOVA) using SAS 9.4 (SAS Institute, Cary, NC, USA). Significant differences between treatment means were analyzed using the Tukey test (p < 0.05). Average values of measurements performed in triplicate were considered for statistical analysis unless otherwise specifically mentioned in any assays.

## 3 Results

### 3.1 Color value of GBR cultivated with illite treatment

A key factor in making food products more appealing to consumers is their color appearance. The current results of the sensory analysis revealed a non-significant difference in the color change with the illite solution treatments in the samples IBR-1, IBR-3, and IBR-5 compared to the control IBR-0 samples Table 1.

### 3.2 Analysis of mineral content of GBR and treated water solution

**Fig. 1:**
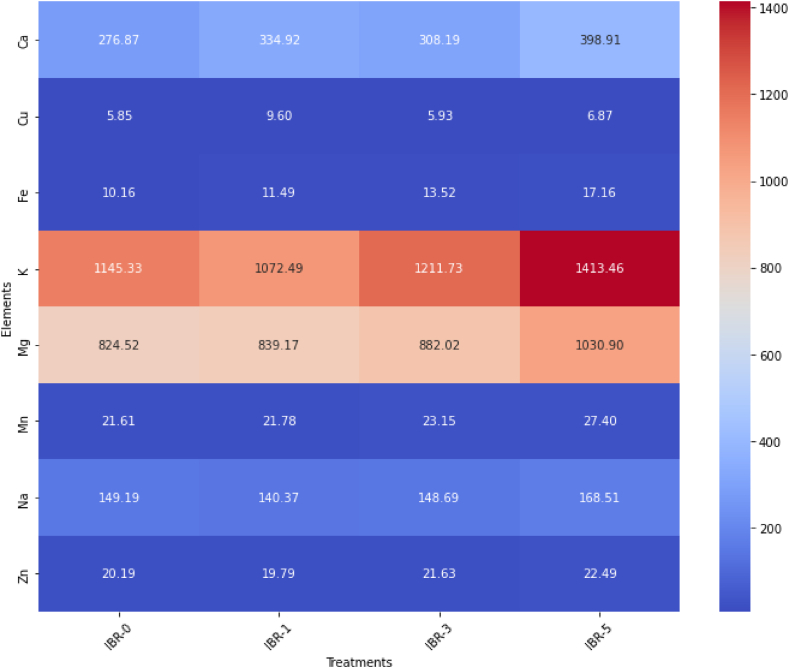
Profiling of mineral elements detected in the germinated brown rice treated with different doses of Illite. The heatmap was created using the heatmap function in RStudio. Quantitative data represents the means±SD.

The mineral content plays a vital role in the growth and development of plants, and their presence is important for human health and diet. Our experiment results showed that after illite treatment, a significant increase in the total mineral content of BR was observed in the IBR-5 (3085.70 mg/kg), followed by the IBR-3 (2614.86 mg/kg) when compared to the control IBR-0 (2453.72 mg/kg). Similarly, among these minerals, a significant improvement was observed in Ca (398.91 mg/kg), Fe (17.16 mg/kg), K (1413.46 mg/kg), Mg (1030.90 mg/kg), Mn (27.40 mg/kg), and Na (168.51 mg/kg) in IBR-5 when compared to the control IBR-0, as shown in Table 2 and Fig. 1. The treatment solution showed no contamination with heavy metals such as As, Hg, Pb, or Cd. The mineral elements such as Ca, Fe, K, Mg, and Na increased with the increased concentration of illite, thus demonstrating the maximum values at a 5 % illite concentration solution. Cu and Zn were not detected in any of the treatments. (Table 3).

**Table 3:** Mineral contents (mg/kg) of distilled water in different concentrations of illite powder.

Element	Sample			
	Control	IBR-1	IBR-3	IBR-5
Ca	24.73 ± 0.08c	20.08 ± 0.02d	26.08 ± 0.06b	35.56 ± 0.06a
Cu	ND	ND	ND	0.01 ± 0.00a
Fe	0.01 ± 0.00d	0.29 ± 0.00c	0.40 ± 0.00b	3.53 ± 0.09a
K	4.08 ± 0.04d	11.56 ± 0.03c	18.40 ± 0.05b	133.07 ± 5.73a
Mg	0.23 ± 0.00c	0.20 ± 0.00d	0.27 ± 0.00b	0.53 ± 0.01a
Mn	ND	0.01 ± 0.00b	0.01 ± 0.00b	0.08 ± 0.00a
Na	19.47 ± 0.00d	29.05 ± 0.09b	23.13 ± 0.17c	41.30 ± 0.25a
Zn	ND	0.01 ± 0.00a	ND	0.01 ± 0.00a
As	ND	ND	ND	ND
Hg	ND	ND	ND	ND
Pb	ND	ND	ND	ND
Cd	ND	ND	ND	ND
Total	48.52	61.20	68.29	214.09

Each data point represents the mean ± standard deviation of at least two replicates. Values followed by different letters in the same row represent significant difference at p < 0.05 (Tukey test). IBR1, IBR2, and IBR3 indicate the water containing 1 %, 2 %, and 3 % (*w/v*) illite powder respectively, ND: Non-detected.

### 3.3 Free amino acid composition of GBR cultivated by illite treatment

**Fig. 2:**
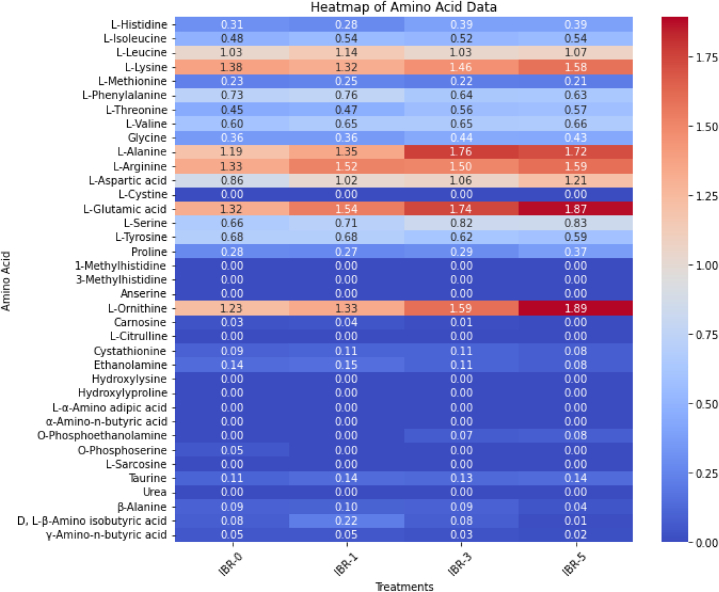
Heatmap indicating the level of amino acids in germinated brown rice when treated with Illite. The heatmap was created using the heatmap function in RStudio. The quantitative value represent the average of at least two replicates.

The free amino acid contents in the BR were significantly increased in the IBR-5 treatment, followed by IBR-3 and IBR-1. Among the essential amino acids, l-lysine, l-isoleucine, l-histidine, and l-threonine showed higher contents in the IBR-5 treatment. A similar trend was observed in IBR3 and IBR1 (Table 4 and Fig. 2). Among the non-essential amino acids, glycine, l-alanine, l-aspartic acid, and l-glutamic acid were significantly increased in the IBR-5 and IBR-3 treatments when compared with the control IBR-0 (Table 4). As a result, the total amino acid and the proportion of essential to non-essential amino acids was considerably enhanced in the IBR-5 treatment, as shown in Table 4 and Fig. 1.

**Table 4:** Free amino acid composition (mg/g of dry weight) of germinated brown rice cultivated after soaking the seeds in different concentrations of illite.

Amino Acid	Sample			
	IBR-0	IBR-1	IBR-3	IBR-5
**Essential Amino Acid**				
**l****-Histidine**	0.31 ± 0.03b	0.28 ± 0.02b	0.39 ± 0.03a	0.39 ± 0.03a
**l****-Leucine**	1.03 ± 0.02b	1.14 ± 0.03a	1.03 ± 0.03b	1.07 ± 0.05b
**l****-Isoleucine**	0.48 ± 0.01b	0.54 ± 0.03a	0.52 ± 0.04a	0.54 ± 0.02a
**l****-Lysine**	1.38 ± 0.02b	1.32 ± 0.01c	1.46 ± 0.07a	1.58 ± 0.07a
**l****-Phenylalanine**	0.73 ± 0.01b	0.76 ± 0.02a	0.64 ± 0.02c	0.63 ± 0.04c
**l****-Methionine**	0.23 ± 0.01a	0.25 ± 0.02a	0.22 ± 0.01a	0.21 ± 0.05a
**l****-Valine**	0.60 ± 0.01b	0.65 ± 0.02a	0.65 ± 0.03a	0.66 ± 0.03a
**l****-Threonine**	0.45 ± 0.02b	0.47 ± 0.03b	0.56 ± 0.01a	0.57 ± 0.02a
**Total**	5.21	5.41	5.47	5.65
**Non-essential Amino Acid**				
**Glycine**	0.36 ± 0.02b	0.36 ± 0.01b	0.44 ± 0.02a	0.43 ± 0.02a
**l****-Arginine**	1.33 ± 0.05b	1.52 ± 0.06a	1.50 ± 0.04a	1.59 ± 0.05a
**l****-Alanine**	1.19 ± 0.03c	1.35 ± 0.07b	1.76 ± 0.05a	1.72 ± 0.06a
**l****-Aspartic acid**	0.86 ± 0.01c	1.02 ± 0.02b	1.06 ± 0.03b	1.21 ± 0.03a
**l****-Glutamic acid**	1.32 ± 0.03d	1.54 ± 0.08c	1.74 ± 0.06b	1.87 ± 0.05a
**l****-Cystine**	ND	ND	ND	ND
**l****-Tyrosine**	0.68 ± 0.02a	0.68 ± 0.02a	0.62 ± 0.02b	0.59 ± 0.05b
**l****-Serine**	0.66 ± 0.01c	0.71 ± 0.01b	0.82 ± 0.01a	0.83 ± 0.06a
**Proline**	0.28 ± 0.02b	0.27 ± 0.01b	0.29 ± 0.01b	0.37 ± 0.03a
**Sub-total**	9.68	7.45	8.23	8.61
**Essential/Non-Essential proportion**	0.54	0.73	0.66	0.66
**Other Free Amino Acid**				
**1-Methylhistidine**	ND	ND	ND	ND
**3-Methylhistidine**	ND	ND	ND	ND
**Anserine**	ND	ND	ND	ND
**l****-Ornithine**	1.23 ± 0.61d	1.33 ± 0.03c	1.59 ± 0.06b	1.89 ± 0.06a
**Carnosine**	0.03 ± 0.01 ab	0.04 ± 0.01a	0.01 ± 0.01b	ND
**l****-Citrulline**	ND	ND	ND	ND
**Cystathionine**	0.09 ± 0.01a	0.11 ± 0.02a	0.11 ± 0.01a	0.08 ± 0.02a
**Ethanolamine**	0.14 ± 0.02a	0.15 ± 0.01a	0.11 ± 0.01b	0.08 ± 0.01c
**Hydroxylysine**	ND	ND	ND	ND
**Hydroxylyproline**	ND	ND	ND	ND
**α-Amino-*n*-butyric acid**	ND	ND	ND	ND
**l****-α-Amino adipic acid**	ND	ND	ND	ND
**O-Phosphoethanolamine**	ND	ND	0.07 ± 0.01a	0.08 ± 0.02a
**O-Phosphoserine**	0.05 ± 0.02a	ND	ND	ND
**l****-Sarcosine**	ND	ND	ND	ND
**Taurine**	0.11 ± 0.03a	0.14 ± 0.01a	0.13 ± 0.02a	0.14 ± 0.01a
**Urea**	ND	ND	ND	ND
**β-Alanine**	0.09 ± 0.01a	0.10 ± 0.01a	0.09 ± 0.01a	0.04 ± 0.02b
**γ-Amino-*n*-butyric acid**	0.05 ± 0.01a	0.05 ± 0.01a	0.03 ± 0.01 ab	0.02 ± 0.01b
**D,****l****-β-Amino isobutyric acid**	0.08 ± 0.01b	0.22 ± 0.02a	0.08 ± 0.01b	0.01 ± 0.01c
**Sub-total**	1.82	2.09	2.19	2.32
**Total Free Amino Acid**	13.76	15.00	15.92	16.60

Values represent mean ± standard deviation of at least two replicates. Values followed by different letters in the same row are significantly different (p < 0.05, analysis of variance (ANOVA), Tukey test). ND: Non-detected.

### 3.4 Total dietary fiber, γ-oryzanol, and phytic acid content of GBR cultivated with illite treatment

The present results show that the total dietary fiber content of BR was significantly improved by the IBR-1 treatment, which gradually decreased with the increase in illite concentration, whereas the IBR-5 treatment showed a significant decrease of 18.1 % dietary fiber content compared to the control IBR-0 treatment (Table 5). In the case of the γ-oryzanol content, IBR-5 treatment showed a significant increase of 87 % compared to the control IBR-0, followed by IBR-3 (31 %) and IBR-1 (18 %) (Table 5). Similarly, changes in the phytic acid content of BR after treatment with different illite concentrations showed a significant increase of 71 % in the IBR-5 treatment, followed by the IBR-3 (45 %) and IBR-1 (35 %) treatments when compared with the control IBR-0.

**Table 5:** Total dietary fiber, γ-oryzanol, and phytic acid content of germinated brown rice cultivated after soaking the seeds in different concentrations of illite.

Sample	Total dietary fiber (g/100g dry weight)	γ-Oryzanol (mg/100g dry weight)	Phytic acid (mg/g dry weight)
**IBR-0**	4.115 ± 0.002b	10.187 ± 0.113d	11.33 ± 0.34c
**IBR-1**	4.702 ± 0.003a	12.098 ± 0.243c	15.56 ± 0.27b
**IBR-3**	4.006 ± 0.009c	14.559 ± 0.190b	16.11 ± 0.74b
**IBR-5**	3.885 ± 0.004d	19.085 ± 0.249a	19.39 ± 0.48a

Each data point represent the mean ± sd of at least two replicates. Different letters in the same column express significant difference at p < 0.05, analysis of variance (ANOVA), Tukey test.

### 3.5 DPPH, SOD-like activity, total polyphenol, and flavonoid contents of germinated brown rice cultivated by illite treatment

The obtained data revealed that illite treatment significantly increased the antioxidant potential of BR (Table 6). The results showed a non-significant difference in the DPPH content in all of the treatments when compared to the control IBR-0. However, the SOD inhibition activities were significantly enhanced in the IBR-5 treatment by 42.1 % compared to the control IBR-0, followed by the IBR-1 treatment, while the IBR-3 treatment showed non-significant differences compared to the control (IBR-0) (Table 6). Furthermore, a moderate difference was found in the phenolic content with the IBR-5 treatment when compared to other and control (IBR-0) treatments, while the IBR-1 and IBR-3 treatments showed a non-significant difference when compared to the control (IBR-0) treatment. Similarly, in the flavonoid contents, the IBR-5 and IBR-3 treatments significantly reduced the TFC by 17.6 % and 8.9 %, respectively, compared to the control (IBR-0), while the IBR-1 treatment showed a non-significant difference compared to the control (Table 6).

**Table 6:** DPPH and superoxide dismutase (SOD)-like activities and total polyphenol and flavonoid contents of germinated brown rice cultivated after soaking the seeds in different concentrations of illite.

Sample	% Inhibition		Total Polyphenol (μg GAE/g dry weight)	Total Flavonoid (μg QE/g dry weight)
	DPPH	SOD-Like Activity		
**IBR-0**	68.77 ± 0.71a	45.45 ± 2.31c	79.35 ± 1.45a	37.39 ± 2.32a
**IBR-1**	69.01 ± 0.52a	49.32 ± 1.55b	79.11 ± 0.81a	36.65 ± 0.09a
**IBR-3**	69.02 ± 0.96a	44.68 ± 2.27c	79.39 ± 1.60a	34.98 ± 0.46b
**IBR-5**	69.02 ± 0.71a	70.80 ± 2.01a	80.18 ± 1.50 ab	28.85 ± 0.09c

QE: quercetin equivalents. GAE: gallic acid equivalents. Each data point represent the mean ± standard deviation of four replicates. Different letters in the same column express significant difference at p < 0.05, analysis of variance (ANOVA), Tukey test.

### 3.6 Effect of soaking on textural properties of BR

**Fig. 3:**
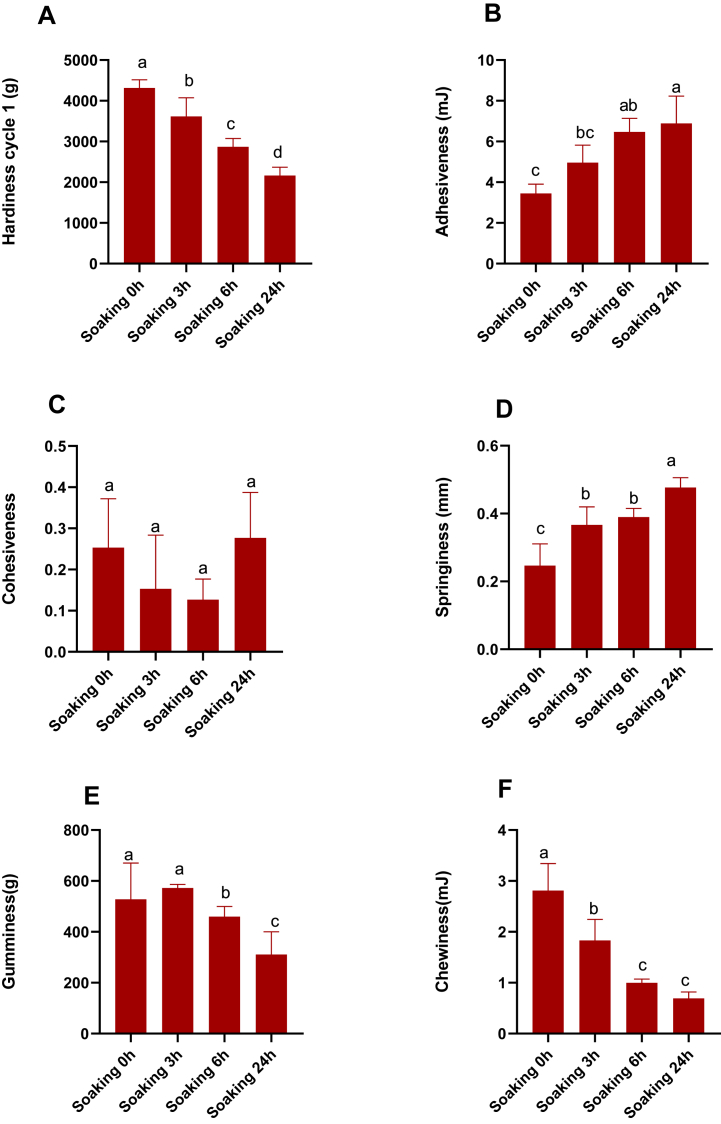
Effect of Illite treatment on textural properties (A) Hardiness, (B) Adhesiveness (C) Cohesiveness (D), Gumminess (E), and Chewiness (F) of germinated brown rice when soaked for different durations. Each data point represents the mean of at least three replicates. Bars with different letters are significantly different from each other at *P* ≤ 0.05.

It was observed that the textural properties of the BR such as hardiness cycle, were significantly reduced with an increase in the soaking duration. However, the chewiness, gumminess, resilience, and springiness were also considerably increased after soaking whereas the cohesiveness remained stable. The textural properties of BR soaked for 6 and 24 h showed only minor differences in several parameters; therefore, soaking for 6 h was determined to be optimal, as shown in Fig. 3.

## 4 Discussion

Improving the nutritional content and texture of BR by soaking is crucial during germination and for consumption [27]. Pretreatment is a reliable method to improve seeds' nutritional content because it possesses several beneficial effects on germination and growth. In the previous experiment by Ha et al. [17], it was discovered that treatment with illite could significantly improve the germination and quality of soybean sprouts without any harmful effects. Based on these findings, we pre-treated BR with illite to enhance its nutritional aspects. We identified that the optimal conditions for the sprouting of BR used in our experiment were soaking for 6 h and cultivation for 48 h, followed by dipping in the same treatment solution for 1 h/4h interval during the germination process at approximately 30 °C. Because BR is a significant source of nutrients, soaking is an important pre-step before cooking and preparing other products to increase its color appearance, nutrient content, and textural properties. In our experiments, we observed that the BR cultivated under the optimal conditions, as mentioned above, showed reduced hardness, and increase of chewiness, gumminess, springiness, as well as stable cohesiveness. This process of preserving the quality of BR and improving its nutritional content may play a significant role in improving the taste and human diet.

Illite generally possesses several mineral elements that could be beneficial to improving seed nutrients and human health. Mineral elements are reported to be involved in energy metabolism, respiration, oxygen transport, DNA synthesis, RNA transcription, cellular growth, and treating various diseases [[28], [29], [30]], and our experiments showed that treatment with illite enhanced the mineral elements of BR, especially with the 3 % and 5 % treatments. It could be presumed that the increase in nutritional content is because of the absorption of the elements that are present in illite. The role of mineral elements in crop development, seed germination, and improved sprout quality is well-reported by several authors [[31], [32], [33]]. Our results are in agreement with Xu et al. [34] and Zou [35], who showed that Zn fortification improved soybean sprout growth and nutritional content. Similar results were demonstrated by Pongrac et al. [36], who reported that the application of natural mineral-rich water improved the nutritional elements and bioactive compounds in wheat sprouts and Tartary wheat. Similarly, sucrose treatment increased GABA, antioxidant activity, micronutrients, and free sugars in Vietnamese GBR [37]. Se enrichment in BR improved its antioxidant stability, protein digestibility, and long-term storage quality [38,39]. With the addition of K, the flavonoids, polyphenolic compounds, vitamin C, and antioxidant capacity were increased in the nutraceutical component basil [40]. Moreover, our results are in line with those of Lee et al. [41], who showed that treatment with illite boosted the antioxidant activity in tomato leaves.

The color of a food product plays a major role in its commercialization, especially for attraction to the consumers' eye as well as in aesthetics in the food industry [42,43]. In our experiment, we noted that there was no color degradation with the illite treatment. There are several articles where the authors applied the extracts of plants and chemicals that have a potential risk of degrading the color appearance of the treated products. However, illite is a nonsticky powder; therefore, while soaking with illite, the mineral elements might penetrate the seeds and improve their mineral content properties. Because of its nonsticky nature, the powder does not attach and change the color of the outer surface of the rice. These results add further importance and scope of the illite treatment in BR. Furthermore, our results revealed that illite treatment improved the amino acid and antioxidant such as flavonoids, polyphenols, and DPPH content of GBR, which varied according to the illite concentration. In addition, γ-oryzanol was also significantly increased, especially with the 3 % and 5 % illite treatments. These findings corroborate with those of several authors, such as Tyagi et al. [44], who reported that slightly acidic electrolyzed water treatment in BR enhanced the antioxidant activity, amino acids, and phenolic and flavonoid contents.

Amino acids are a source of crude proteins for several whole grains, such as wheat, oats, barley, and rye, including BR [45]. Treatment with external elements may exert stress on a crop, which may result in oxidative stress, thereby disrupting its antioxidants, amino acid pathway, and protein composition. For example, treatment with high levels of Se has an inhibitory effect on amino acid synthesis in soybeans [46], and salinity exposure induces polymorphism in quinoa seeds [47].

Moreover, phenolics play a crucial role in the amino acid biosynthetic pathways, such as the phenylpropanoid pathway [48]. Our experiment revealed that illite treatments could strengthen polyphenol, SOD levels, and amino acid content. Our results are supported by the findings of Sritongtae et al. [49], who showed that pretreatment of ricebean with citric acid enhanced the phenolic content, radical-scavenging activity, and amino acid (alanine, glutamic acid, and serine) content. Chitosan, salicylic acid, and H_2_O_2_ treatments increased the phenolic and flavonoid contents of the Dalia bean [50]. In addition, Kamjijam et al. [51] reported that amino acids, such as glutamine, glutamic acid, and lysine, were increased after 72 h of germination in rice var. Riceberry and improved its palatability. Because our results showed that the content of essential elements, such as Ca, K, Fe, and Mg, were higher in the illite solution-treated groups, it is likely that the penetration of these elements into the seeds played a crucial role in regulating the amino synthesis in the sprouts. Moreover, the germination type and time may have a significant influence on the amino acid synthesis and composition [52].

Antioxidants such as phenolics and flavonoids are concentrated in the aleurone layer, bran, and germ of BR [53,54]. Antioxidants and amino acid biosynthesis are interlinked phenomena in crop metabolism that are primarily involved in cross-signaling [55]. Ionic interplay has a vital role in plant protection from decay, oxidation, and activating the antioxidative activities in seeds and crops. Mineral elements regulate the amino acid synthesis and ionic balance, thereby improving the metabolomics of several crops and human health [[56], [57], [58]]. Because illite treatment improved the polyphenol content and preserved the flavonoids, the high concentrations of minerals such as Ca and P in illite might have played a key role in the oxidative protection, germination, and improving the quality of GBR [59,60]. From these results, we predict that treatment with the appropriate illite concentration could maintain the amino acid threshold levels for industrial and dietary purposes.

During grain germination, certain macromolecules are destroyed through the synthesis of new cellular components, which can considerably improve the sensory and nutritional characteristics of the cereal [61]. Various hydrolases are activated to hydrolyze the proteins and carbohydrates in cereals during the germination process, which improves the *in vitro* digestibility and flavor of the germinated BR. Furthermore, germination can enrich the bioactive substances, including γ-oryzanol, phenolics, GABA, minerals, dietary fiber, and minimize the phytic acid, a potential antinutrient component [37,62]. In general, the phytic acid level is decreased during the germination process in BR. In our study, the phytic acid was considerably reduced in the control (with water only); however, an increase in phytic acid was observed with an increase in the illite treatment. In our experiment, the decrease in phytic acid in BR upon germination in the control is corroborated by several authors, including Cáceres [63] and Liang et al. [64]. The increase in phytase activity may be related to the reduced phytic acid levels in GBR during the germination process [65]**.** Therefore, it is crucial to determine the appropriate concentration of illite that could maintain the nutritional and qualitative properties without hampering the taste of the BR-derived food. A phenomenon was reported by Zhao et al. [66] where the increase of phytic acid in BR could be associated with the phytase and phosphatase reaction and acidification that hydrolyze phytic acid and activate the exogenous and endogenous phytase and phosphatase in BR.

Moreover, our study showed that the dietary fiber was considerably enhanced in the IBR-1 treatment, while the increase in illite concentration reduced the dietary fiber content of GBR. It has been reported that the dietary fiber content and composition could fluctuate depending on the germination method and conditions [67,68]. According to the report by Mohan et al. [69], the dietary fiber content of BR was significantly increased by germination. Contrastingly, a report by Jayadeep and Malleshi [70] indicated that the germination of BR by soaking, followed by atmospheric conditions, reduced the dietary fiber content. A similar trend was observed by Kim et al. [67], where atmospheric germination reduced the dietary fiber content.

Some precautions should be considered while soaking BR and treating it with illite [71]. A higher illite concentration may increase the phytate and decrease the dietary fiber, which may degrade the nutritional value and sprout yield [71]. Additionally, the optimal soaking time and temperature are crucial to improving palatability and contamination from microorganisms to prevent the outbreak of foodborne illness [72]. Moreover, the animal experiment test is required to ensure the safety of Illite before employing it for commercial production.

## 5 Conclusion

Overall, our findings revealed that the appropriate concentration of illite pretreatment of seeds could be beneficial because it does not hamper germination or induce oxidative stress but rather enhances several beneficial characteristics such as antioxidant activities, mineral element content, and γ-oryzanol without affecting the color or amino acid degradation. Therefore, illite can be considered a potent and safe element for seed treatment. Further experiments are required for its commercial application, which is in operation.

## Funding statement

This study was financially supported by the Research & Development Illite project of Yeongdong-gun in Chuncheongbuk-do Province, Korea.

## Data availability statement

The dataset supporting the findings of this study will be made available from the corresponding author upon reasonable and justified request.

## CRediT authorship contribution statement

**Dong-Heun Han:** Supervision. **Hwa-Jin Kim:** Methodology. **So-Hyun Kim:** Investigation. **Il-Doo Kim:** Project administration. **Arjun Adhikari:** Writing – review & editing, Investigation. **Jeong-Ho Kim:** Conceptualization.

## Declaration of competing interest

The authors declare that they have no known competing financial interests or personal relationships that could have appeared to influence the work reported in this paper.
