# Supplementary material for: Effect of illite pretreatment on germinated Brown rice with Special Reference to amino acids, antioxidants, texture, and mineral elements
Source: Heliyon. 2024 Apr 9;10(8):e28843. doi: 10.1016/j.heliyon.2024.e28843 (PMC11043867; doi:10.1016/j.heliyon.2024.e28843)
Supplement: Multimedia component 1 [file mmc1.docx]

| **Time Period** | **Soaking 0h** | **Soaking 3h** | **Soaking 6h** | **Soaking 24h** |
| --- | --- | --- | --- | --- |
| **DAY 1** |  | **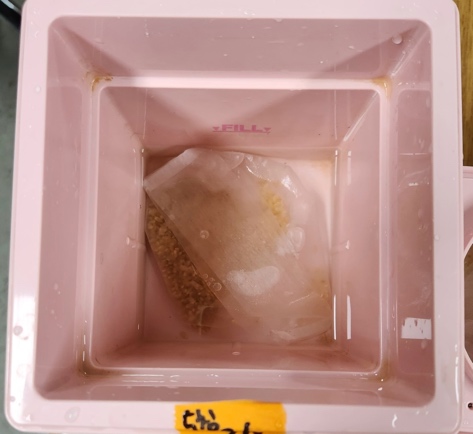** | **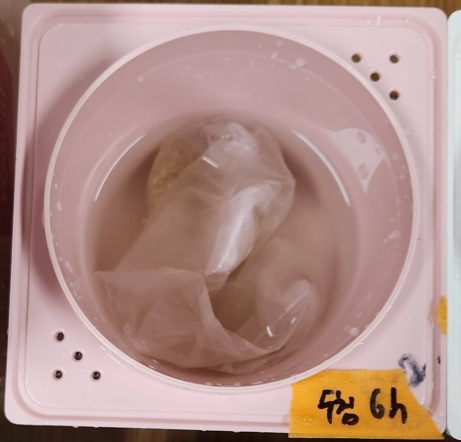** | **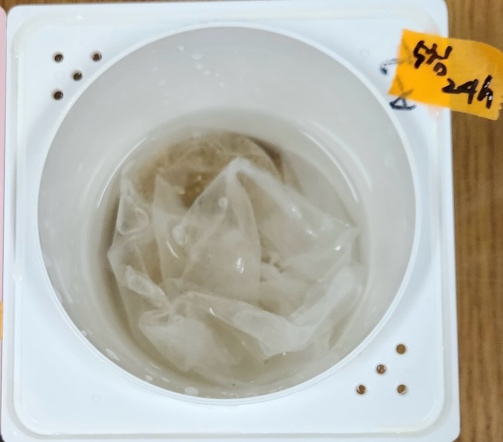** |
| **Day 2** | **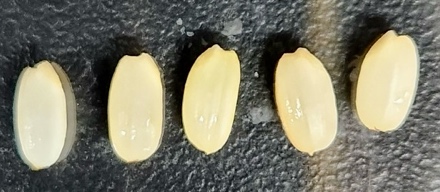** | **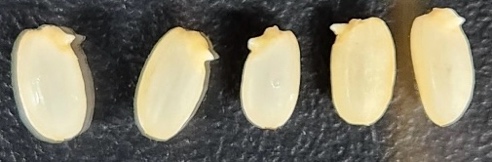** | **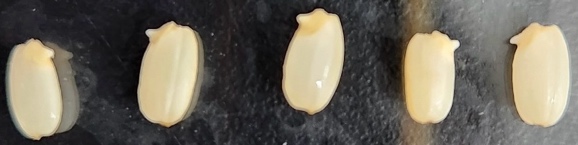** | **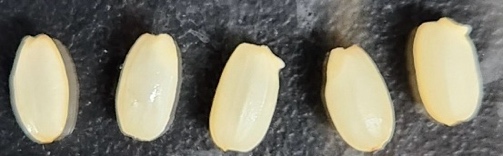** |

**Supplementary Table 1: Time Table indicating the germination condition to determine the optimal growth condition.**

| **Time Period** | **Soaking 0h** | **Soaking 3h** | **Soaking 6h** | **Soaking 24h** |
| --- | --- | --- | --- | --- |
| **DAY 3** | **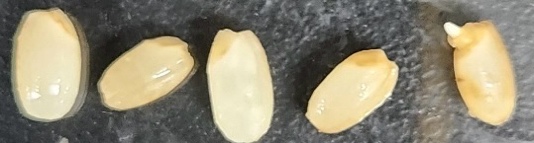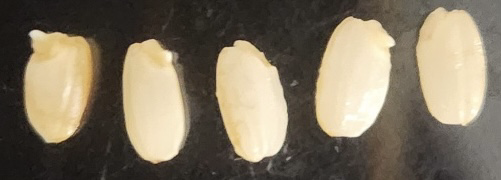** | **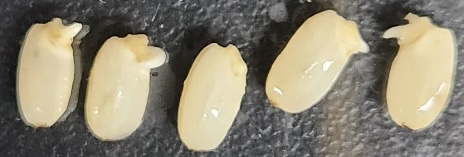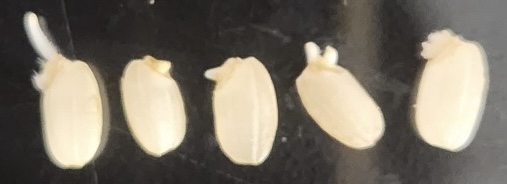** | **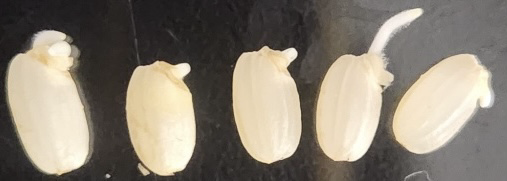**  9.A.M  **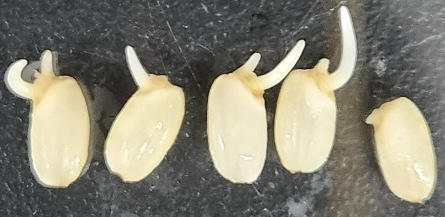**  5 P.M. | **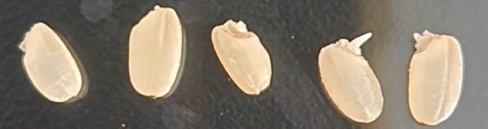** |
| **DAY 4** |  |  |  | **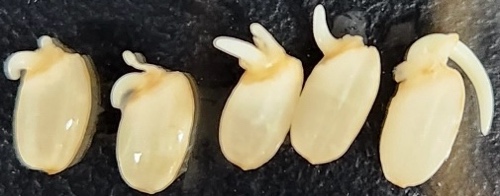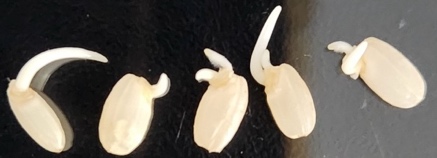** |

**Supplementary Table 2: Time Table indicating the optimal germination condition of brown rice cultivation with 1% Illite treatment**

| **Time Period** | **(Soaking 6h)** | **Cultivation 48h**  **(Soaking 6h)** | **Cultivation 72h**  **(Soaking 6h)** |
| --- | --- | --- | --- |
| **Day 1** | **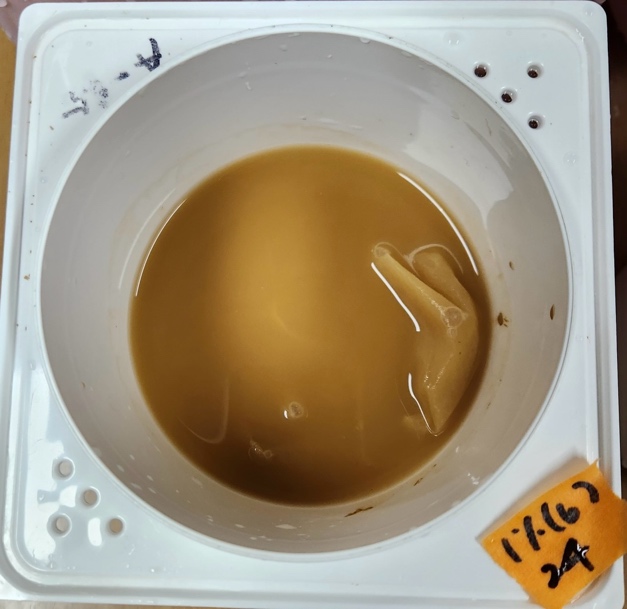** | **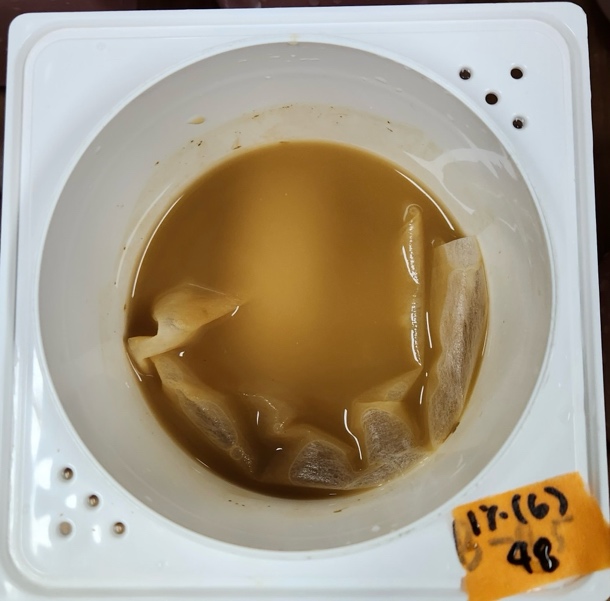** | **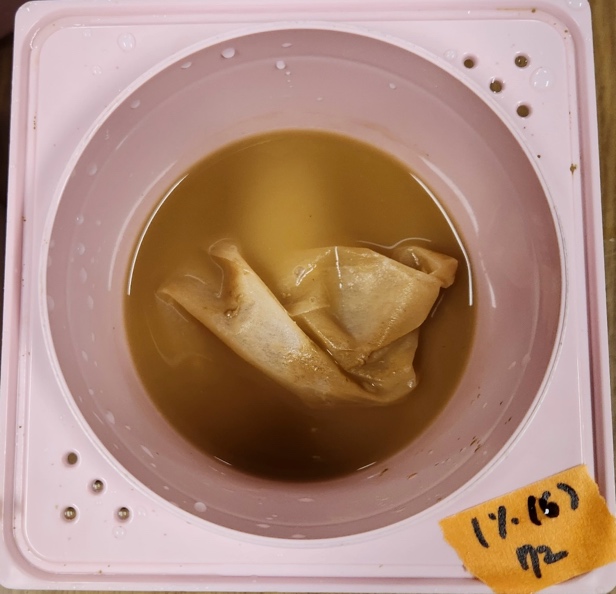** |
| **DAY 2** | **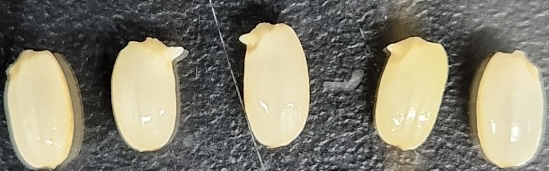**  **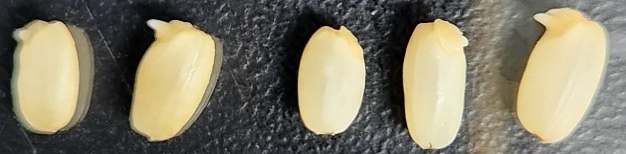** | **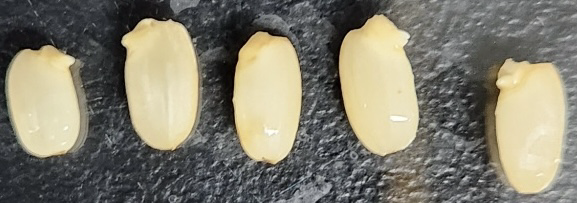** | **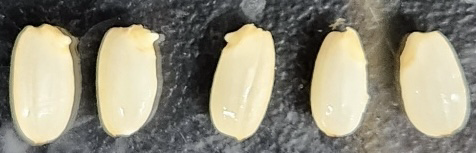** |

|  | **Cultivation 24h**  **(Soaking 6h)** | **Cultivation 48h**  **(Soaking 6h)** | **Cultivation 72h**  **(Soaking 6h)** |
| --- | --- | --- | --- |
| **DAY 3** |  | **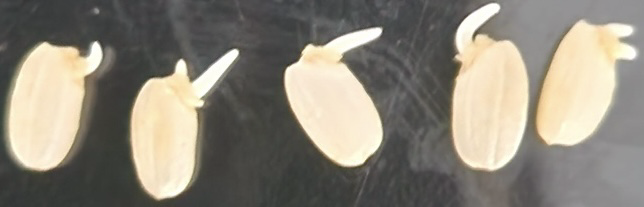**  (9 A.M)  **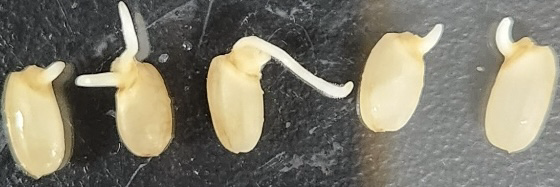**  (5 P.M.) | **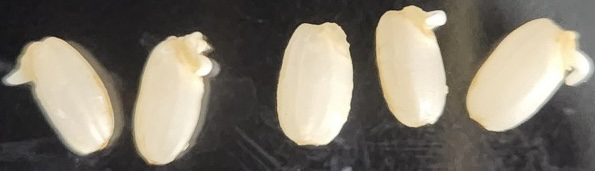** |
| **DAY 4** |  |  | **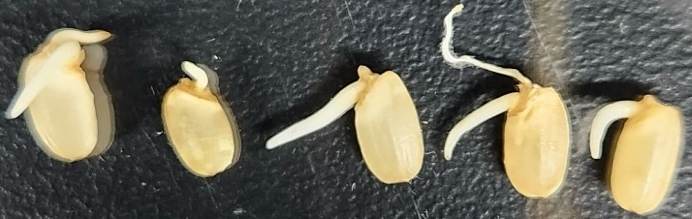**  **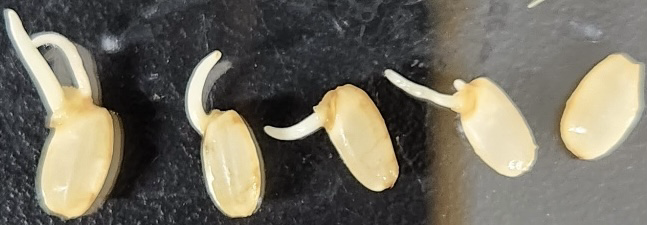** |
